# Pubertal and adult windows of susceptibility to a high animal fat diet in *Trp53-null* mammary tumorigenesis

**DOI:** 10.18632/oncotarget.13112

**Published:** 2016-11-04

**Authors:** Yirong Zhu, Mark D. Aupperlee, Yong Zhao, Ying Siow Tan, Erin L. Kirk, Xuezheng Sun, Melissa A. Troester, Richard C. Schwartz, Sandra Z. Haslam

**Affiliations:** ^1^ Cell and Molecular Biology Program and Breast Cancer and the Environment Research Program, Michigan State University, East Lansing, MI, USA; ^2^ Department of Physiology and Breast Cancer and the Environment Research Program, Michigan State University, East Lansing, MI, USA; ^3^ Department of Microbiology and Molecular Genetics and Breast Cancer and the Environment Research Program, Michigan State University, East Lansing, MI, USA; ^4^ Department of Epidemiology, University of North Carolina at Chapel Hill, NC, USA; ^5^ Lineberger Comprehensive Cancer Center, University of North Carolina at Chapel Hill, NC, USA; ^6^ Department of Pathology and Laboratory Medicine, University of North Carolina at Chapel Hill, NC, USA

**Keywords:** dietary animal fat, breast cancer, puberty, adulthood, Trp53-null

## Abstract

Premenopausal breast cancer is associated with increased animal fat consumption among normal weight, but not overweight women (Farvid et al., 2014). Our previous findings in obesity-resistant BALB/c mice similarly showed promotion of carcinogen-induced mammary tumorigenesis by a diet high in saturated animal fat (HFD). This effect was specific to pubertal versus adult HFD. This study identifies the effects of HFD during puberty versus adulthood in *Trp53-null* transplant BALB/c mice and investigates its mechanism of enhancing tumorigenesis. Either pubertal or adult HFD is sufficient to increase incidence of *Trp53-null* mammary tumors. Puberty-restricted HFD exposure promoted tumor cell proliferation, increased angiogenesis, and increased recruitment of total and M2 macrophages in epithelial tumors. Adult-restricted exposure to HFD similarly increased proliferation, angiogenesis, recruitment of total and M2 macrophages, and additionally reduced apoptosis. Adult HFD also increased incidence of spindle cell carcinomas resembling claudin-low breast cancer, and thus adult HFD in the *Trp53-null* transplantation system may be a useful model for human claudin low breast cancer. Importantly, these results on *Trp53-null* and our prior studies on DMBA-induced mammary tumorigenesis demonstrate a pubertal window of susceptibility to the promotional effects of HFD, indicating the potential of early life dietary intervention to reduce breast cancer risk.

## INTRODUCTION

Effective prevention strategies are needed to combat breast cancer. Lifestyle modifications, especially changes in diet, have been heavily investigated as potential preventative measures. Some studies reported that a western diet, rich in saturated fat, is associated with increased breast cancer risk [1]. However, meta-analyses examining the association of total fat and saturated fat intake with breast cancer risk are inconsistent, partly because of differences in study design, dietary classification, dietary assessment at the time of cancer diagnosis, and varied baseline breast cancer incidence among the diverse populations studied [2–6]. The interaction of fat intake with breast cancer risk may also be subtype specific [7]. While high fat diet (HFD) often leads to obesity, a recent analysis of the Nurses' Health Study II identified consumption of a high animal fat diet in early adulthood to increase pre-menopausal breast cancer risk only in normal weight women, suggesting that dietary effects in tumor promotion may be obesity independent [8].

Human and rodent models have demonstrated that the mammary gland is sensitive to environmental and dietary influences during puberty [9, 10]. We previously reported that life-long exposure to HFD initiated in puberty compared to a life-long low fat diet (LFD) [11] or HFD restricted to puberty [12] similarly reduced the latency of 7,12-dimethylbenz[a]anthracene (DMBA)-induced tumors. Mice overexpressing *HER2/neu* had increased development of second tumors when HFD was introduced in pubertal mice at 4 weeks of age [13], but tumor incidence was not affected when HFD was introduced to adult mice at 10 weeks of age [14], again suggesting the importance of timing in HFD exposure. In this regard, the human epidemiological study by Linos et al. [15] specifically indicates adolescent exposure to total dietary fat as a risk factor for premenopausal breast cancer, while not finding a significant association with subtypes of fat.

DMBA must be introduced to mice during puberty to efficiently initiate mammary tumors. In our prior studies [12], some of the effects of pubertal HFD observed in the pre-tumor mammary gland were only evident if the mice also received pubertal DMBA exposure. To circumvent the potential confounding interaction of pubertal HFD with pubertal DMBA exposure, the present study investigated the effects of pubertal versus adult exposure to HFD on mammary tumorigenesis in obesity resistant BALB/c mice, using the *Trp53-null* transplantation model. *Trp53* is one the most frequently mutated genes in human breast cancer [16, 17]. We found in this model that both pubertal and adult life stages were susceptible to the promotional effects of HFD. Puberty-restricted HFD exposure promoted tumor cell proliferation, increased angiogenesis, and increased recruitment of total and M2 macrophages in epithelial tumors, similarly to adult-restricted exposure to HFD. Adult HFD exposure uniquely increased the occurrence of estrogen and progesterone receptor negative (ER- PR-), poorly differentiated spindle cell carcinomas. These findings further implicate HFD as a risk factor in the occurrence of mammary cancer.

## RESULTS

### Either pubertal or adult exposure to HFD promotes tumorigenesis

**Figure 1:**
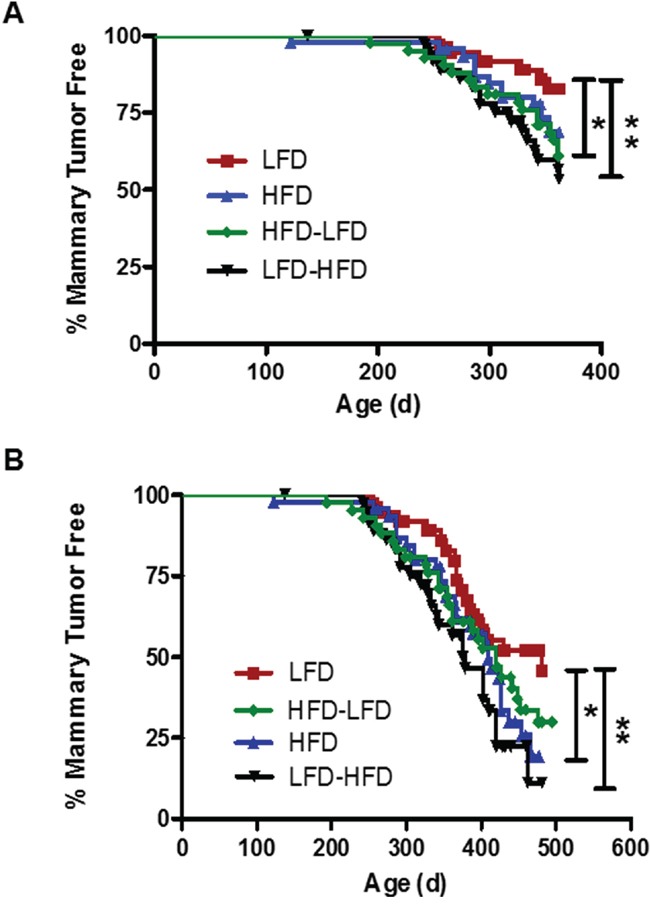
The effect of the various dietary regimens on the number of tumor-free mammary glands over time Kaplan-Meier plots were determined for BALB/c mice receiving *Trp53-null* mammary transplants and fed a lifelong LFD (LFD; n= 37 glands), a lifelong HFD (HFD; n= 46), a puberty-restricted HFD (HFD-LFD, n=42), and an adult-restricted HFD (LFD-HFD, n=38). **A.** The number of tumor-free mammary glands over one year was significantly reduced in mice fed HFD-LFD and LFD-HFD. (Log-rank tests, LFD vs. HFD, p = 0.158; LFD vs. HFD-LFD, *, p = 0.042; LFD vs. LFD-HFD, **, p = 0.009). **B.** The number of tumor-free mammary glands over 500 days was significantly reduced by HFD and LFD-HFD, but not HFD-LFD. (Log-rank tests, LFD vs. HFD, *, p = 0.046; LFD vs. HFD-LFD, p = 0.131; LFD vs. LFD-HFD, **, p = 0.006).

In our previous studies in the DMBA mammary tumorigenesis model [11, 12], we observed key differences in the properties of early versus late occurring tumors in response to dietary regimen. Kaplan-Meier analysis revealed that the one-year mammary tumor incidence in mice receiving *Trp53-null* mammary transplants (Figure 1A) was significantly increased by puberty-restricted HFD (HFD-LFD, 39%; 2.2-fold; p=0.042), as well as by adult-restricted HFD (LFD-HFD, 47%; 2.6-fold; p=0.009) compared to LFD (17%). Continuous HFD also caused a 1.7-fold increase in tumor incidence by one year of age, but the difference did not reach statistical significance (HFD 31%; p=0.16). Following tumor development up to 500 days of age at the end of the study (Figure 1B) showed tumor incidence was increased by continuous HFD (HFD, 81%; 1.8-fold; p=0.046) and adult-restricted HFD (LFD-HFD, 89%; 2-fold; p=0.006) compared to continuous LFD (54%); the tumor promotional effects of puberty-restricted HFD (HFD-LFD, 70%; 1.6-fold; p=0.13) were less dramatic when viewed over the longer time course and also showed a trend toward increased tumor incidence. Kaplan-Meier analysis found no significant differences in tumor latency by diet treatments (data not shown).

### Tumor characteristics

**Figure 2:**
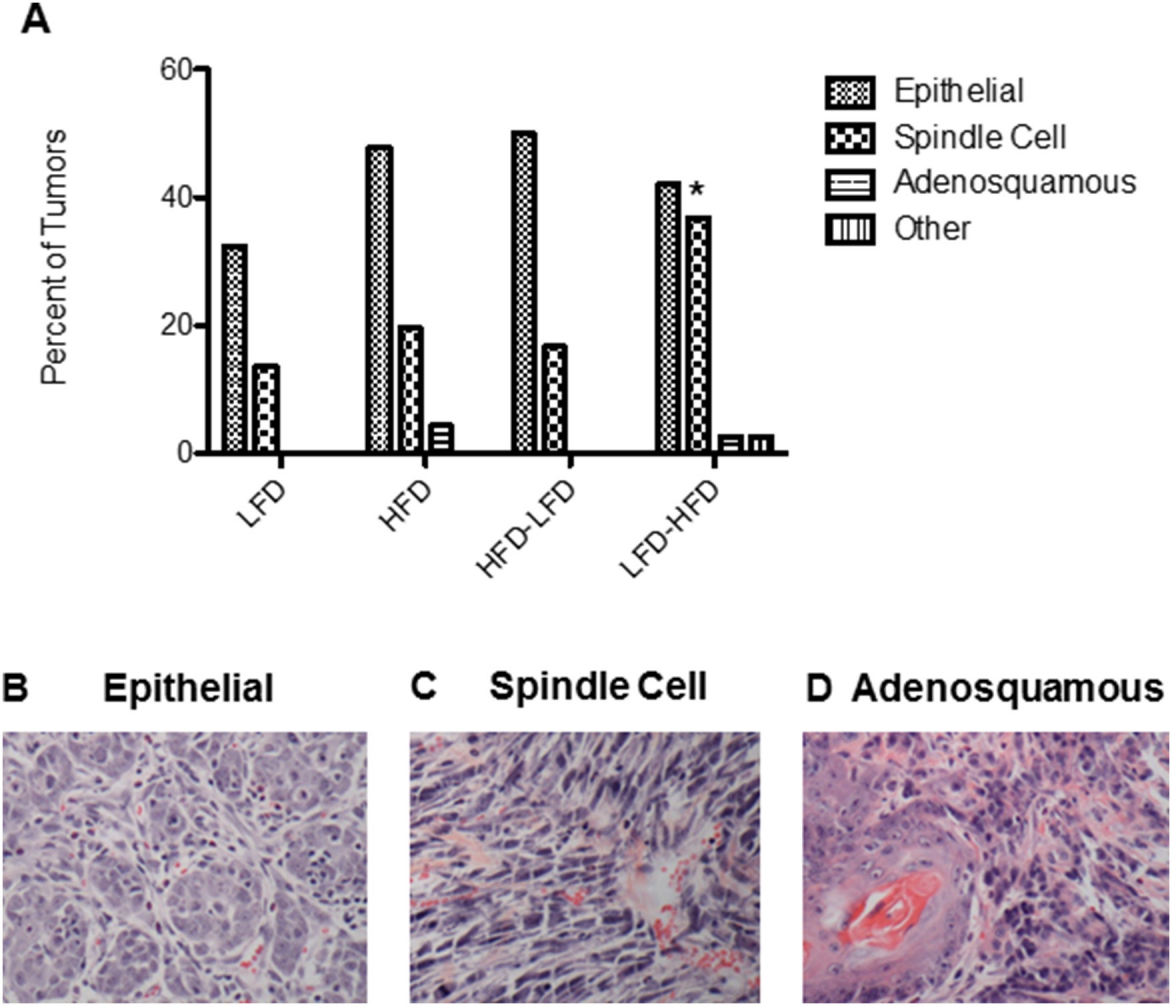
Proportions of tumors by histopathology across the different dietary regimens in BALB/c mice receiving *Trp53-null* mammary transplants **A.** LFD-HFD increased the proportion of spindle cell carcinomas by 2.7-fold compared to LFD (p = 0.02). **B-D.** Representative H&E stained sections of epithelial, spindle cell and adenosquamous tumors. Scale bar = 0.25mm.

Tumors of multiple histopathologic types (Figure 2) developed in *Trp53-null* mammary transplants. Regardless of the diet treatment, the majority of tumors were epithelial in composition. However, some of the tumors were poorly differentiated spindle cell carcinomas. LFD-HFD mice had a significantly increased number of spindle cell carcinomas per transplant compared to mice on other dietary regimens (p=0.02) (Figure 2A). The majority of the epithelial tumors (63-82% among the dietary regimens) were ER- PR- and did not vary significantly by histological type or diet treatment, and all spindle cell tumors were ER- PR- (Supplementary Table S1). The majority of the spindle cell tumors also lacked expression of keratins 5 and 18, markers of luminal differentiation (data not shown).

In quantifying various properties of the tumors in the current study, in addition to comparisons between tumors arising under the four dietary regimens, we compared early (<52 weeks latency; mean latency 42 weeks, 6.7 weeks SD) versus later occurring (≥52 weeks; mean latency 59 weeks, 4.3 weeks SD) epithelial tumors. This separation into early versus late developing tumors distinguished between the time period where mice fed HFD-LFD showed significantly higher tumor incidence and the longer time period where significance was not observed (see Figure 1). We also compared epithelial tumors to spindle cell tumors. For the latter group, we did not distinguish between early and late occurring tumors because of the limited number of tumors.

**Figure 3:**
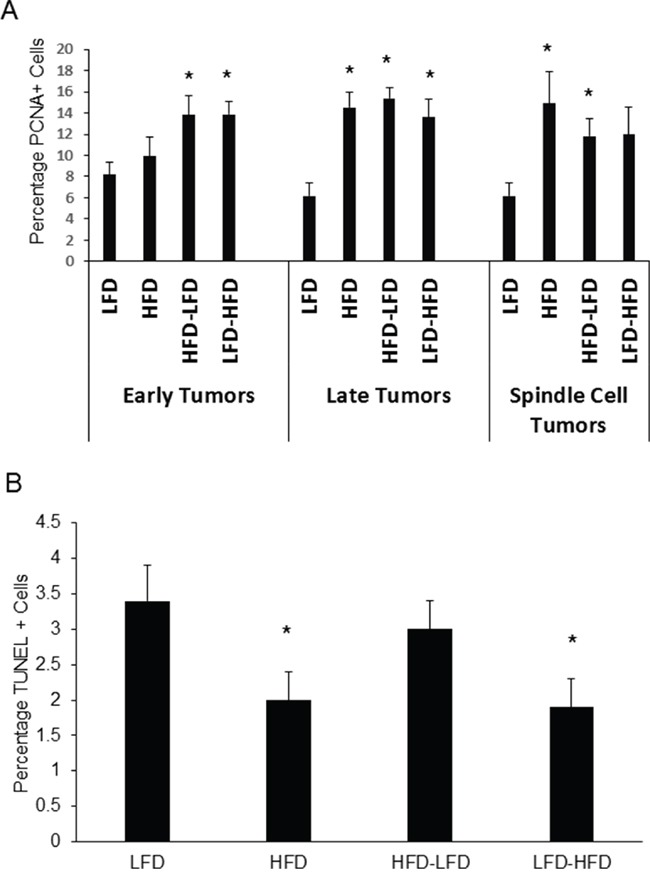
Effects of various dietary regimens on tumor proliferation and apoptosis **A.** HFD at any life stage tended to increase expression of the proliferation marker PCNA in late occurring epithelial and spindle cell tumors (*, p < 0.05). LFD versus LFD-HFD in spindle cell tumors, p = 0.067. LFD versus HFD in early occurring epithelial tumors was not significant. Tumors are grouped by epithelial and spindle cell histology. Epithelial tumors are additionally grouped by latency: early tumors had latencies less than one year; late tumors had latencies of one year or greater. Early tumors: LFD (n=5); HFD (n=8); HFD-LFD (n=6); LFD-HFD (n=8). Late tumors: LFD (n=5); HFD (n=5); HFD-LFD (n=6); LFD-HFD (n=3). Spindle cell tumors: LFD (n=5); HFD (n=5); HFD-LFD (n=6); LFD-HFD (n=6). **B.** Lifelong HFD (HFD) and adult-restricted HFD (LFD-HFD), irrespective of either latency or histopathology, decreased apoptosis as measured by TUNEL labeling (*, p < 0.05). LFD (n=15); HFD (n=18); HFD-LFD (n=18); LFD-HFD (n=17).

Since uncontrolled proliferation and escape from apoptosis are hallmarks of cancer, we measured tumor cell proliferation and apoptosis by quantifying nuclear localization of proliferating cell nuclear antigen (PCNA) and terminal deoxynucleotidyl transferase dUTP nick end labeling (TUNEL), respectively. ANOVA (Supplementary Table S2) found that dietary effects on proliferation were significant, whether examining both epithelial and spindle cell tumors or epithelial tumors alone. While time of epithelial tumor onset and identification as a spindle cell tumor were not significant in regard to proliferation, a significant interaction was found between diet and time of epithelial tumor onset. HFD, HFD-LFD, and LFD-HFD mice all exhibited significantly increased proliferation in late occurring epithelial tumors (2.2 to 2.6-fold) and HFD and HFD-LFD spindle cell tumors (1.6 to 2.0-fold) compared to tumors from LFD mice (Figure 3A). In early occurring epithelial tumors, increased proliferation was observed for HFD-LFD and LFD-HFD mice, but not for HFD mice. To see if enhanced proliferation was specific to tumor cells, we also measured the effect of diet on proliferation in normal *Trp53-null* transplanted tissue. No significant effects on proliferation were observed in normal tissue of 19-week old mice, prior to the appearance of any tumors (data not shown). ANOVA (Supplementary Table S2) found that dietary effects on apoptosis were significant when examining both epithelial and spindle cell tumors. An interaction between diet and time of epithelial tumor onset approached significance (p=0.09). Apoptosis was significantly decreased in HFD and LFD-HFD mice (Figure 3B).

**Figure 4:**
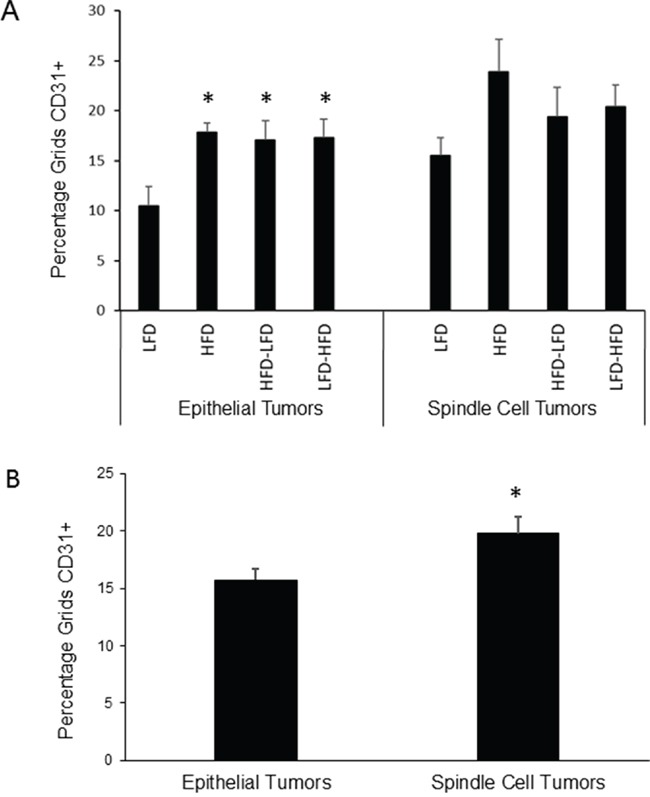
Effects of dietary regimens on tumor vascularity **A.** Among epithelial tumors, HFD at any life stage tended to increase blood vessel density as measured by CD31 staining (*, p<0.05). No significant effects were observed among spindle cell tumors. Epithelial tumors: n=8 for each dietary group. Spindle cell tumors: n=4 for each dietary group. **B.** Spindle cell tumors were more vascularized than epithelial tumors (*, p<0.05). Epithelial tumors: n=32; Spindle cell tumors: n=16.

Because HFD was associated with increased angiogenesis in DMBA-induced tumors, we analyzed intra-tumoral vascularization with the endothelial cell marker, CD31. ANOVA (Supplementary Table S2) found that dietary effects on vascularization were significant, whether examining both epithelial and spindle cell tumors or epithelial tumors alone. Further, time of epithelial tumor onset and identification as a spindle cell tumor were also significant in regard to vascularization, but epithelial tumor onset by itself was not significant, indicating that spindle cell tumors differed from epithelial tumors. No significant interaction was observed between diet and time of epithelial tumor onset and identification as a spindle cell tumor. Epithelial tumors on all regimens that included HFD showed significant 1.6 to 1.7-fold increased vascularization compared to mice fed LFD (Figure 4A). Among spindle cell tumors, only those occurring in HFD mice showed an increase in vascularization that approached significance (p=0.06). Spindle cell tumors showed significant 1.3-fold increased vascularization compared to epithelial tumors (Figure 4B). No significant effects on vascularity were observed in normal tissue of 19-week old mice, prior to the appearance of any tumors (data not shown).

**Figure 5:**
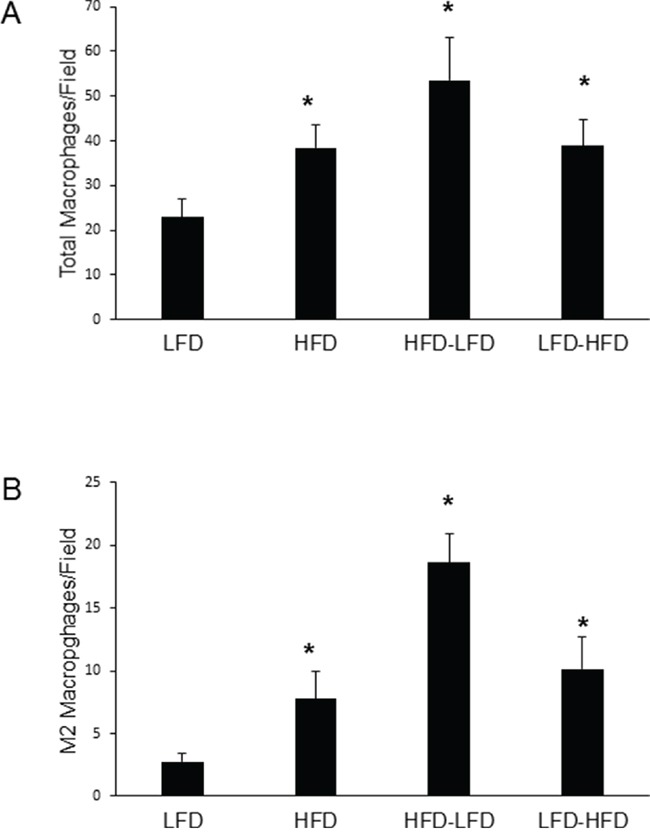
Quantitation of macrophages within epithelial tumors **A.** HFD at any life stage increased total macrophages as measured by F4/80 staining (*, p<0.05). **B.** HFD at any life stage increased M2 macrophages as measured by F4/80-Arg1 double staining (*, p<0.05). For both panels A and B, LFD (n=9); HFD (n=9); HFD-LFD (n=8); LFD-HFD (n=8).

Macrophages play important roles in normal mammary development and tumorigenesis [18, 19]. We examined both intra-tumor macrophage (F4/80+) localization and the extent of M2 (i.e., Arginase 1 positive (Arg1+)) polarization in these macrophages. ANOVA (Supplementary Table S2) found that dietary effects on both total and M2 macrophage levels were significant, whether examining both epithelial and spindle cell tumors or epithelial tumors alone. While time of epithelial tumor onset and identification as a spindle cell tumor were not significant in regard to macrophage levels, an interaction approaching significance was found between diet and time of epithelial tumor onset for total macrophages (p=0.09). Among epithelial tumors, significantly increased numbers of total (Figure 5A) and M2 (Figure 5B) macrophages were observed under all diet regimens that included a period of HFD exposure compared to continuous LFD. There were no significant dietary effects among spindle cell tumors (data not shown). The majority of macrophages were Arg1- (i.e., likely M1, classically activated) in all tumors across all diet treatments (data not shown). While HFD-related increases in total macrophages ranged from 1.7 to 2.3-fold, it is noteworthy that increases in M2 macrophages ranged from 2.8 to 6.8-fold (HFD-LFD: 6.8-fold; LFD-HFD: 3.7-fold; HFD: 2.8-fold). At 19-weeks of age, HFD-LFD mice showed a 1.9-fold increase (p=0.091) in the number of macrophages in normal mammary gland over that found for LFD mice (Supplementary Figure S1). The overwhelming majority (85 to 91%) of these macrophages were of a M2 phenotype (data not shown).

### Microarray analysis of gene expression in tumors

The tumor samples from the individual dietary regimens did not show significant gene expression changes according to specific dietary regimen, due to small sample size (n=8 for epithelial tumors, n=3 for spindle cell carcinomas; data not shown). Considering pubertal HFD exposure, a microarray analysis comparing HFD + HFD-LFD versus LFD + LFD-HFD tumors (false discovery rate (FDR)=0.10) identified 55 upregulated genes (GEO: GSE74294), but no significant canonical pathways or molecular functions were identified (data not shown). Comparison of early occurring epithelial tumors to late occurring epithelial tumors (FDR=0.10), irrespective of diet, identified a number of differentially regulated molecular functions (Supplementary Table S3). The three most statistically significant were “Immunological Disease: systemic autoimmune syndrome” (p=9.18E-13), “Endocrine System Disorders, Gastrointestinal Disease, Immunological Disease, Metabolic Disease: insulin-dependent diabetes mellitus” (p=8.51E-14), and “Endocrine System Disorders, Gastrointestinal Disease, Metabolic Disease: diabetes mellitus” (p=9.18E-13). Comparison of spindle cell tumors to the various epithelial tumors (FDR=0.05) revealed 1594 significantly upregulated and 1840 significantly downregulated genes (GEO: GSE74294), but no significant canonical pathways were identified and, while some significantly upregulated molecular functions were identified, none of these molecular functions had levels of significance approaching those of molecular functions identified in the comparison of early to late epithelial tumors (data not shown).

### Dietary effects on metabolic parameters

**Figure 6:**
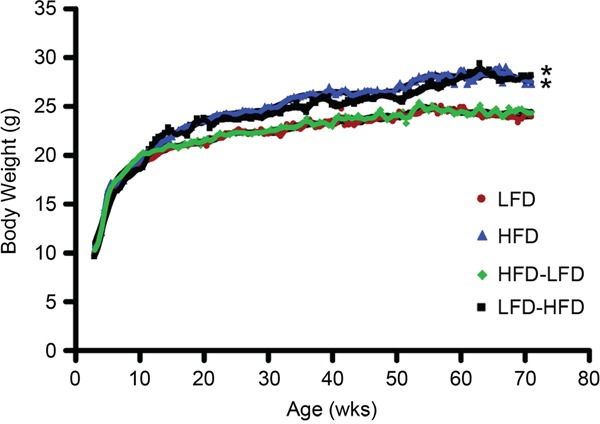
Body weight over time Mice fed lifelong HFD had increased body weight compared to mice fed lifelong LFD after 7 weeks on diet, while adult-restricted HFD mice (LFD-HFD) had increased body weight 7 weeks after their diet was switched to HFD. Puberty-restricted HFD mice (HFD-LFD) had similar weights to those fed lifelong LFD. The weight gains in mice fed HFD and LFD-HFD were 14±8 % and 17±9 %, respectively, by end of the experiment at 71 weeks of age. LFD mice (n=33); HFD mice (n=31); HFD-LFD (n=31); LFD-HFD (n=31).

**Figure 7:**
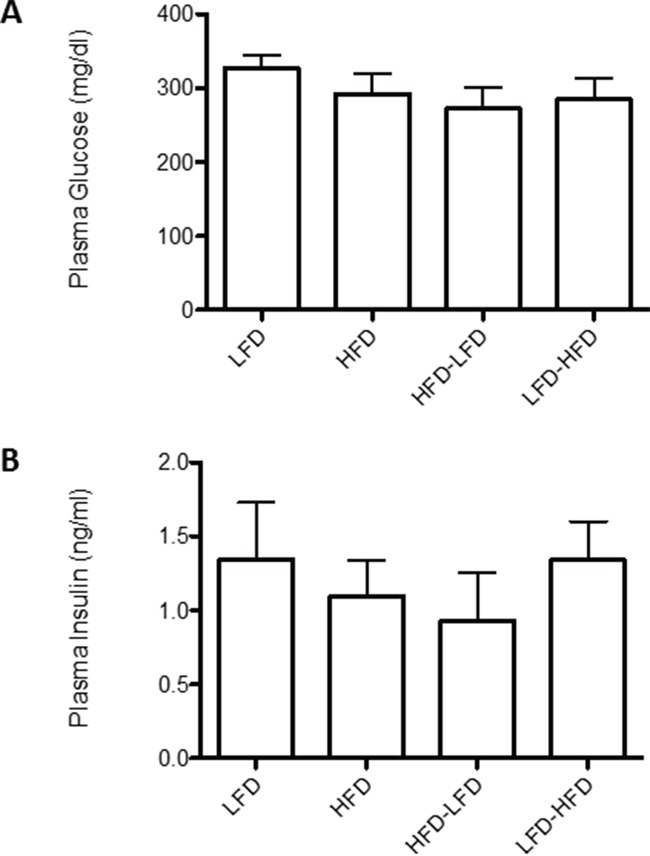
Effects of dietary regimens on plasma levels of glucose and insulin Non-fasting plasma **A.** glucose and **B.** insulin levels were measured in tumor bearing mice. No significant differences were found between dietary regimens. LFD (n=8); HFD (n=8); HFD-LFD (n=8); LFD-HFD (n=8).

BALB/c mice were previously reported to be obesity resistant on HFD [11]. In the present study, we observed modest weight gain in HFD and LFD-HFD mice (Figure 6). However, the weight gain was neither correlated with altered non-fasting plasma glucose levels (Figure 7A) nor non-fasting plasma insulin levels (Figure 7B) in tumor-bearing mice. As the mice aged, there was a trend towards increased plasma glucose levels, but not plasma insulin levels, in all diet groups (data not shown). In 19-week old mice, prior to the appearance of any tumors, glucose and insulin levels were similar between dietary groups (Supplementary Figure S2).

## DISCUSSION

In *Trp53-null* initiated tumors, we have identified two different life stage periods of increased tumor development in response to HFD. Puberty-restricted HFD (HFD-LFD) was sufficient to increase tumor incidence in mice at one year of age, but the magnitude of this promotional effect diminished over a longer time frame. Despite switching to LFD in adulthood, the short 7 weeks of pubertal HFD exposure promoted tumor cell proliferation, increased angiogenesis, and increased recruitment of total and M2 macrophages in epithelial tumors, similarly to adult-restricted exposure to HFD.

*Trp53-null* initiated tumors were also promoted by adult-restricted HFD. This differs from our findings on HFD exposure in the DMBA model, where pubertal exposure was required for enhanced tumorigenesis [11, 12]. It is possible that this difference has a basis in *Hras1* being a common mutational target in DMBA-induced mammary tumorigenesis [20–22], while *Trp53-null* mutations may reduce the occurrence of *Hras1*-mutant tumors [23]. It is also plausible that the longer time course of tumorigenesis for the *Trp53-null* tumors simply provides a longer window for adult HFD effects to occur than does the shorter window for adult HFD exposure in the DMBA model. Over a longer 500-day time frame, adult-restricted HFD and lifelong HFD initiated in puberty significantly increased tumor incidence. Among epithelial tumors, in addition to promoting tumor cell survival by reducing apoptosis, lifelong and adult-restricted HFD also enhanced angiogenesis, and increased recruitment of total and M2 macrophages. Unlike puberty-restricted HFD exposure, lifelong exposure to HFD did not increase tumor cell proliferation. In addition to increasing overall mammary tumor incidence, only adult-restricted HFD, among the dietary regimens, significantly increased the incidence of spindle cell carcinoma, which was previously characterized to have many features of epithelial-to-mesenchymal transition (EMT) and a gene expression profile similar to the claudin-low (CL) intrinsic subtype of human breast cancer [24]. About 65-80% of CL human breast cancers are reported to be ER- PR- and lacking expression of keratins 5 and 18 [25]. With our *Trp53-null* mammary transplant model, all spindle cell carcinomas were ER- PR-, and the majority lacked keratin 5 and 18 expression. Genomic analysis of CL breast cancer identified a high level of genomic instability with many gains and losses. This suggests that CL tumors are likely driven by several oncogenic events. It is noteworthy that both lifelong and adult-restricted HFD mice experience lengthy exposure to HFD, yet the dramatic increase in spindle cell carcinoma is specific to adult-restricted HFD. Examination of macrophage levels in pre-tumor mammary glands showed that macrophage levels were elevated in mice exposed to a puberty-restricted HFD (i.e., HFD-LFD; p=0.091) and showed a similar trend in mice with lifelong HFD. Perhaps this early increase in the number of macrophages prior to tumor occurrence partially suppresses the occurrence of spindle cell carcinomas under lifelong and puberty-restricted HFD regimens, while adult-restricted HFD mice, having fewer macrophages in pre-tumor tissue, are more permissive for their occurrence.

The defining characteristic of puberty-restricted HFD is heightened recruitment of M2 macrophages to the normal mammary epithelium prior to tumor development. Heightened recruitment to normal epithelium was initially observed in our earlier studies of the effects of puberty-restricted HFD in both the presence of DMBA and in the absence of a tumor-inducing agent [12], and is reiterated in these current studies in the *Trp53*-null mouse model. This may indicate a critical role of macrophages in puberty, as well as the sensitivity of puberty to HFD effects. In this regard, macrophages are indeed known to be essential for pubertal mammary gland development [18]. Furthermore, *Trp53*-null tumors occurring in mice exposed to puberty-restricted HFD show the highest levels of both total and M2 macrophage recruitment among tumors occurring across the several dietary regimens. This was also the case in DMBA-induced mammary tumorigenesis [12]. Tumor-associated M2 macrophages are known to promote the growth of tumors through support of angiogenic and tissue remodeling processes, as well as immune suppression [26]. Their presence at early pre-neoplastic stages in tumor development suggest a critical role in tumorigenesis [27].

The defining characteristic of adult-restricted HFD is heightened occurrence of spindle cell carcinoma, which resembles CL human breast cancers. As noted above, this may be an inverse function of macrophage recruitment. The various dietary regimens did not differentially affect spindle cell carcinoma proliferation, apoptosis, or vascularization. It is also unlikely that the increased incidence of spindle cell carcinoma with adult-restricted HFD is a consequence of the metabolic effects of extended HFD exposure, as similar levels of plasma and glucose were observed across all dietary regimens.

Pathway analysis based on RNA expression of the tumors found that early occurring tumors upregulated molecular functions associated with type 1 diabetes and systemic autoimmune syndrome. The genes identified across these molecular functions are almost completely identical and, while associated with the pathology of diabetes, are genes more generally associated with autoimmune activity. Previous studies have implicated p53 in immune regulation. A functional *Trp53* polymorphism is associated with susceptibility to several autoimmune disorders, including systemic lupus erythematosus [28–31], Hashimoto's thyroiditis [32], and rheumatoid arthritis [33]. Most relevant to the current study, somatic *Trp53* mutations were identified in the synovia of rheumatoid arthritis patients [34–38], pointing to the function of p53 in non-immune cells in the etiology of autoimmunity. A likely mechanism for a *Trp53-null* genotype mediating enhanced autoimmune activity is dysregulation of apoptosis. For example, *Trp53* drives epithelial *CD200* expression, which reduces immune reactivity to apoptotic self-antigens [39]. Another apoptotic related function of *Trp53* is the induction of the pro-apoptotic death domain 1α protein (DD1α), which is involved in macrophage recognition and clearance of apoptotic cells [40]. DD1α-deficient mice show autoimmunity and inflammation associated with their inability to clear apoptotic cells. Consistent with the modulation of apoptosis with age, ANOVA performed on TUNEL values found an interaction between diet and time of epithelial tumor onset that approached significance. Future studies should evaluate whether failure to express *CD200* and/or *DD1*α may be the basis for enhanced autoimmunity under *Trp53*-deficiency. Further, it should be noted that that dietary treatments were largely without effect on glucose and insulin levels, highlighting the immune rather than metabolic significance of the identified molecular functions. It is interesting that increased autoimmune activity is identified in early versus later occurring tumors. An age-related decline in T cell activity is well-established in mouse models (reviewed in [41]). At the same time, there is also an increased abundance of immunosuppressive Treg cells with age (reviewed in [42]).

We previously reported that a puberty-initiated lifelong HFD decreased DMBA-induced tumor latency, partly by increasing tumor proliferation, angiogenesis and recruitment of M2 macrophages [11]. Recently, we reported that only puberty-restricted HFD, but not adult-restricted HFD, promoted DMBA-induced tumors [12]. A similar pubertal sensitivity to HFD was identified in the MMTV-*neu* model, where only puberty-initiated HFD promoted development of second tumors [13]. However, here we find that HFD confers similar changes in proliferation, angiogenesis, and macrophage recruitment to *Trp53-null* initiated tumors, when given either only in puberty or only in adulthood. This discrepancy in the life stage-related effects of HFD between tumor models might be caused by differential sensitivity to HFD between tumor subtypes. Indeed, recent human epidemiological studies suggest that this may be the case in humans [7]. In the DMBA carcinogenesis model, a significant proportion of the early developing tumors from mice fed a puberty-restricted HFD were adenosquamous carcinomas [11, 12], which are rare in the *Trp53-null* transplant model. In contrast, the spindle cell carcinomas promoted by adulthood-restricted HFD in the *Trp53-null* transplant model are rare in other murine carcinogenesis models [24].

A limited number of randomized, controlled studies of dietary intervention to prevent breast cancer have been carried out with mixed conclusions. One study that focused on women with high mammographic density found no reduction in breast cancer risk with reduced dietary fat [43]. Another study focusing on post-menopausal women, while finding no significant protective effect for low fat intervention, reported a trend toward a protective effect in women with highest baseline fat intake [44]. However, these women also showed significant weight loss with low fat intervention, so it is unclear whether the benefit is from low fat in itself or from decreased body mass index (BMI). Here, we found that switching mice from a pubertal HFD to an adult LFD does not confer short-term protective effects. Puberty-restricted HFD mice showed increased incidence of mammary tumors over LFD mice during the initial year of our study. Over a longer time course of 500 days, the difference in incidence of mammary tumors in puberty-restricted HFD and LFD mice became less significant. Whether this reflects a reduction in the risk posed by pubertal HFD or alleviation of adult-specific dietary risk is unclear because adult-restricted HFD poses a significant risk in itself. Nonetheless, the existence of tumor promotional effects by HFD at both puberty and adulthood, and the potential to alleviate the HFD-associated risk demonstrated in this study, offer hope for using dietary intervention as a breast cancer prevention strategy. However, the profound effects of pubertal exposure to HFD on tumor development highlight the importance of successful dietary intervention programs and the need for early life intervention strategies.

Obesity/high BMI (BMI > 30 kg/m^2^) is a well-established risk factor for post-menopausal breast cancer [45]. However, normal weight women who consume high saturated fat and high animal fat diets have increased pre-menopausal breast cancer risk [8]. In the current study, modest weight gains of 14-17% were observed in BALB/c mice fed puberty-initiated lifelong and adult-restricted HFD. This modest weight gain is consistent with previous studies in BALB/c mice demonstrating their resistance to HFD-induced obesity [11, 12, 46]. In the absence of obesity, both puberty- and adult-restricted HFD enhanced tumor cell proliferation [11, 12]. In a similar non-obesogenic context, HFD stimulated growth of injected 4T1 cells in BALB/c mammary fat pads, and promoted liver and lung metastases [47]. Collectively, our findings and those of others suggest that a diet rich in animal fat may promote proliferation in mammary tumors. Our earlier studies found that HFD also stimulated proliferation in normal mammary epithelium [11, 12, 46], however we did not observe this in the current study. The earlier studies examined mice at 7 and 13 weeks of age, while the current study examined normal epithelium at 19 weeks of age. The proliferative response of normal epithelium to HFD may be a property of puberty. We found that mice at 7 and 13 weeks of age have higher basal levels of mammary epithelial proliferation than do mice at 19 weeks of age. Thus, HFD may induce increased proliferation during developmental stages when the mammary epithelium is already proliferating.

These studies show that exposure to HFD restricted to either puberty or adulthood is sufficient to promote *Trp53-null* mammary tumorigenesis in the absence of obesity. This confirms our earlier studies [11, 12] that identified puberty as a life stage particularly sensitive to the promotional effects of HFD in mammary tumorigenesis, and extends these findings to identify adulthood as an independent period of sensitivity to HFD given an appropriate oncogenic initiator. Notably, adult-restricted HFD not only increased the incidence of mammary tumors, but specifically increased the incidence of spindle cell carcinomas that resemble CL breast cancer, suggesting that this may be a useful animal model for human CL breast cancer. Epithelial tumors that developed in mice fed HFD at any life stage showed varying levels of enhanced tumor proliferation, angiogenesis, and macrophage recruitment, all plausible contributors to tumor promotion. Importantly, the identification of puberty as a particularly sensitive life stage for the promotional effects of HFD indicates the potential of an early life dietary prevention strategy to reduce the risk of breast cancer.

## MATERIALS AND METHODS

### Mice

BALB/c *Trp53*^+/−^ breeding mice were obtained from Dr. D. Joseph Jerry (University of Massachusetts, Amherst MA), and *Trp53-null* mice were generated as described [48]. The *Trp53-null* tissue donor mice were maintained on chow diet before mammary gland collection at eight weeks of age. Wild-type recipient female BALB/c mice were purchased from Charles River (Portage, MI) at 3 weeks of age. Mice were randomly assigned into four diet groups (see Diets). Food and water were provided *ad libitum*. Animals were housed in a standard laboratory housing environment with a 12:12 h light–dark cycle, at 20 to 24°C with 40 to 50% relative humidity. All animal experimentation was conducted in accord with accepted standards of humane animal care under guidelines approved by the All University Committee on Animal Use and Care at Michigan State University (AUF #09/14-176-00).

### *Trp53*-null mouse model

Fragments of donor mammary epithelium were collected from female BALB/c *Trp53-null* mice at 8 weeks of age, and transplanted into the cleared inguinal mammary fat pads of 3-week-old female wild type mice as previously described [49, 50]. To minimize donor bias from secondary genetic alterations, mammary duct fragments from 4 donor mice were transplanted to recipient mice in each diet group in equal distribution. Body weights and food consumption were monitored weekly. Animals were palpated for tumor development twice a week starting at 13 weeks of age. Tumors were harvested at 1 cm in diameter. Portions of tumors and mammary glands were formalin-fixed, paraffin embedded for H&E and immunohistochemistry, and the remaining portions of tumors were snap-frozen for later RNA isolation. Mice were monitored for 500 days, and at termination of the studies, mammary glands were formalin-fixed and processed as whole mounts to evaluate transplantation success rate. Transplantation success rates were 59%, 77%, 64% and 72% for the LFD, HFD, LFD-HFD, HFD-LFD groups, respectively. Mammary glands that had no epithelium present were excluded from the analysis of tumor incidence. See Supplementary Table S4 for further detail on mammary gland transplantation and occurrence of mammary tumors.

### Diets

**Figure 8:**
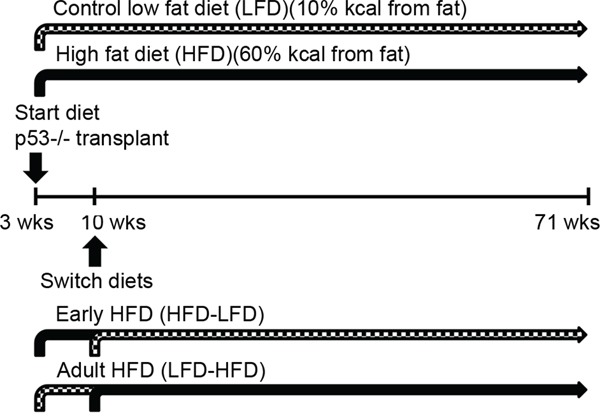
Experimental scheme Fragments of *Trp53-null* mammary duct were transplanted into cleared fat pads of female 3-week-old wild-type BALB/c mice. After the surgery, mice were randomly assigned to four diet groups. The LFD group (n=33) and the HFD group (n=31) were fed their respective diets lifelong and *ad libitum*. To investigate the effects of HFD exposure restricted to puberty, a group of HFD-fed mice (n=31) were switched to LFD (HFD-LFD) at 10 weeks of age. Similarly, to investigate the effects of HFD exposure restricted to adulthood, a group of LFD-fed mice (n=31) were switched to HFD (LFD-HFD) at 10 weeks of age. Tumor development was monitored until mice reached 500 days (71 weeks) of age.

Low fat diet (D11012202; 10% kcal fat) and high fat diet (D11012204; 60% kcal fat) were purchased from Research Diets (New Brunswick, NJ). See Supplementary Table S5 for detailed composition of the diets. For the continuous LFD and HFD groups, diets were initiated after transplantation at 3 weeks of age and maintained throughout the studies. For the HFD-LFD and LFD-HFD groups, mice were initially fed one diet from 3 weeks until 10 weeks of age, and then switched to the other diet thereafter (Figure 8).

### Immunohistochemistry

5 μm tumor sections were deparaffinized and rehydrated, as previously described [11]. Antigen retrieval was accomplished by autoclaving at 121°C and 15 psi for 30 minutes in citrate buffer (pH 6.0). For blood vessel density determinations, CD31 was detected with rabbit polyclonal anti-CD31 (1:50 in PBS–0.5% Triton X-100; Cat #: AP15436PU-N; Acris Antibodies, Inc., San Diego, CA) at RT for 2 hours. Images were captured using a Nikon Eclipse E400 light microscope (Nikon, Inc., Melville, NY) with a 40X objective lens. The images were overlaid with grids containing 240 squares (324 μm^2^/square). Blood vessel density is expressed as the percentage of CD31-positive squares. For proliferation, PCNA was detected using goat polyclonal anti-PCNA (1:100 in PBS–0.5% Triton X-100; Cat #: sc-9857; Santa Cruz, Biotechnology, Inc., Santa Cruz, CA) at 4°C overnight. For macrophage detection, double staining of F4/80 and Arg1 has been described previously [11] using monoclonal rat anti-F4/80 (1:75 in PBS–0.5% Triton X-100; Cat #: MCA497R; AbD Serotec, Raleigh, NC) and goat anti-Arg1 (1:200 in PBS–0.5% Triton X-100; Cat #: sc-18354; Santa Cruz Biotechnology, Inc.). Detection of ERα and PR was performed as previously described [11]. ERα was detected with mouse anti-ERα (1:10 in PBS–0.5% Triton X-100; Cat #: NCL-ER-6F11; Novocastra Laboratories, Ltd, Newcastle upon Tyne, UK) and PR was detected with rabbit anti-PR (1:200 in 2% BSA in phosphate-buffered saline; Cat #: A0092; DAKO, Carpinteria, CA). Cytokeratin 18 (K18) was detected with mouse anti-cytokeratin 18 (1:75 in PBS–0.5% Triton X-100; Cat #ab668; Abcam, Cambridge, MA) and cytokeratin 5 (K5) with rabbit anti-cytokeratin 5 (1:500 in PBS–0.5% Triton X-100; Cat # 905501; BioLegend, San Diego, CA). Immunofluorescent staining was completed with appropriate secondary antibodies. All immunofluorescence sections were counterstained with 4',6-diamidino-2-phenylindole (DAPI) to visualize nuclei. Images were captured with a Nikon Eclipse TE2000-U fluorescence microscope (Nikon, Inc.) using a 40x objective. At least 1000 cells and at least 3 sections per animal were analyzed. For proliferation, cells were scored positive with the presence of speckled nuclear localization of PCNA [51]. Macrophage density was expressed as the number of F4/80 positive cells per tumor image. For ERα and PR assessment, a minimum of 1000 cells were counted for each tumor. Tumors were considered to be ERα+ if 10% or more of the total cells counted were ERα+ [52]; a minimum of 500 cells per section for each tumor were counted.

### TUNEL

5μm tumor sections were deparaffinized and rehydrated. TUNEL analysis was performed using the TdT-FragEL DNA Fragmentation Detection Kit (EMD Millipore, Billerica, MA) following the manufacturer's directions. At least 1000 cells and at least 3 sections per animal were analyzed.

### Metabolic parameters

Plasma glucose and insulin levels were measured from samples collected at sacrifice from non-fasting tumor-bearing animals, as previously described [11]. Plasma glucose levels were determined by OneTouch UltraMini (LifeScan, Inc., Milpitas, CA) and the insulin levels were determined with the rat/mouse insulin ELISA kit (EMD Millipore), according to the manufacturer's instructions.

### Microarray analysis

Agilent Technologies (Santa Clara, CA) 4X44K whole mouse genome microarrays were performed according to manufacturer protocol with linear amplification and 2-color hybridization using total RNA isolated from mouse mammary tumors (Supplementary Table S6). The reference channel was Universal Mouse Reference (as described in [53]) and was labeled with Cy5. Spots that had intensity greater than 10 dpi in at least 80% of samples were selected for subsequent analysis. Data were Lowess normalized and missing data were imputed using k-nearest neighbors with k = 10. A total of 41 microarrays were analyzed. Two class Significance Analysis of Microarrays was performed to identify differentially expressed genes between early vs. late tumor onset among all tumors and among spindle cell and epithelial cell carcinomas separately, pubertal HFD vs. LFD, and spindle cell vs. epithelial carcinoma. All statistical analyses were conducted in R using the samr package in Bioconductor. The cutoff for significance was a 0.10 false discovery rate (FDR) for early occurring epithelial tumors versus late occurring epithelial tumors and for HFD + HFD-LFD versus LFD + LFD-HFD tumors. The cutoff for significance was a 0.05 FDR for spindle cell tumors versus epithelial tumors. A pre-set FDR threshold is generally not advised because the analysis may result in huge variations in the number of candidate genes [54]. An appropriate signature size is important in empirical analyses, since either too many or too few genes may not provide informative and reliable biological interpretations. Based on this consideration, we relaxed the FDR threshold for analysis of early versus late tumors and for HFD + HFD-LFD versus LFD + LFD-HFD tumors to capture more potential biological information; while in the analysis of epithelial versus spindle cell tumors, where thousands of significant genes were identified, a more stringent threshold was used to reduce the number of false positive genes. For genes significantly associated with early versus late tumor onset, gene ontology analyses were conducted using Ingenuity Pathway Analysis. All microarray data discussed in this publication have been deposited in the National Center for Biotechnology Information Gene Expression Omnibus (GEO) [55] database and are accessible at accession number [GEO: GSE74294] [Puberty-specific promotion of mammary tumorigenesis by a high animal fat diet in P53 −/− mice http://www.ncbi.nlm.nih.gov/geo/query/acc.cgi?acc=GSE74294 Access date: 24 Oct 2015].

### Statistical analysis

Results are shown as mean ± standard error of the mean (SEM). Differences were considered significant at p <0.05 using Student's t-test or analysis of variance (ANOVA) followed by the Tukey multiple comparison test, as appropriate. Mammary tumor free survivals were determined from Kaplan-Meier plots by log-rank tests. Tumor incidence was analyzed by the Chi-square test.

## SUPPLEMENTARY FIGURES AND TABLES




