# Supplementary material for: Pubertal and adult windows of susceptibility to a high animal fat diet in Trp53-null mammary tumorigenesis
Source: Oncotarget. 2016 Nov 4;7(50):83409–23. doi: 10.18632/oncotarget.13112 (PMC5347778; doi:10.18632/oncotarget.13112)
Supplement: Supplementary file 1 [file oncotarget-07-83409-s001.pdf]

Pubertal and adult windows of susceptibility to a high animal fat diet in *Trp53-null* mammary tumorigenesis

SUPPLEMENTARY FIGURES AND TABLES

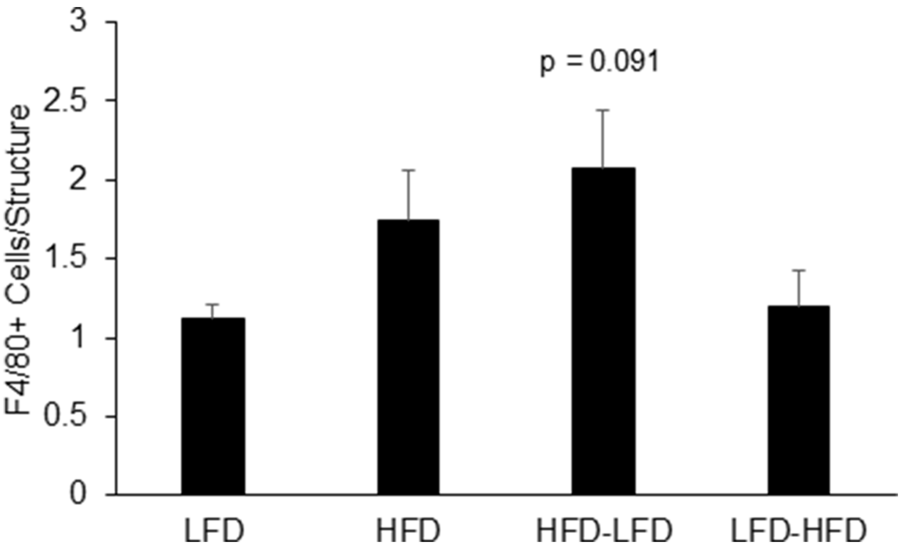

Supplementary Figure S1: Recruitment of macrophages to normal mammary glands of 19-week old mice across dietary regimens. The increase in macrophages in the normal mammary glands of HFD-LFD mice compared to LFD mice approached significance. LFD (n=8); HFD (n=4); HFD-LFD (n=4); LHF-HFD (n=4).

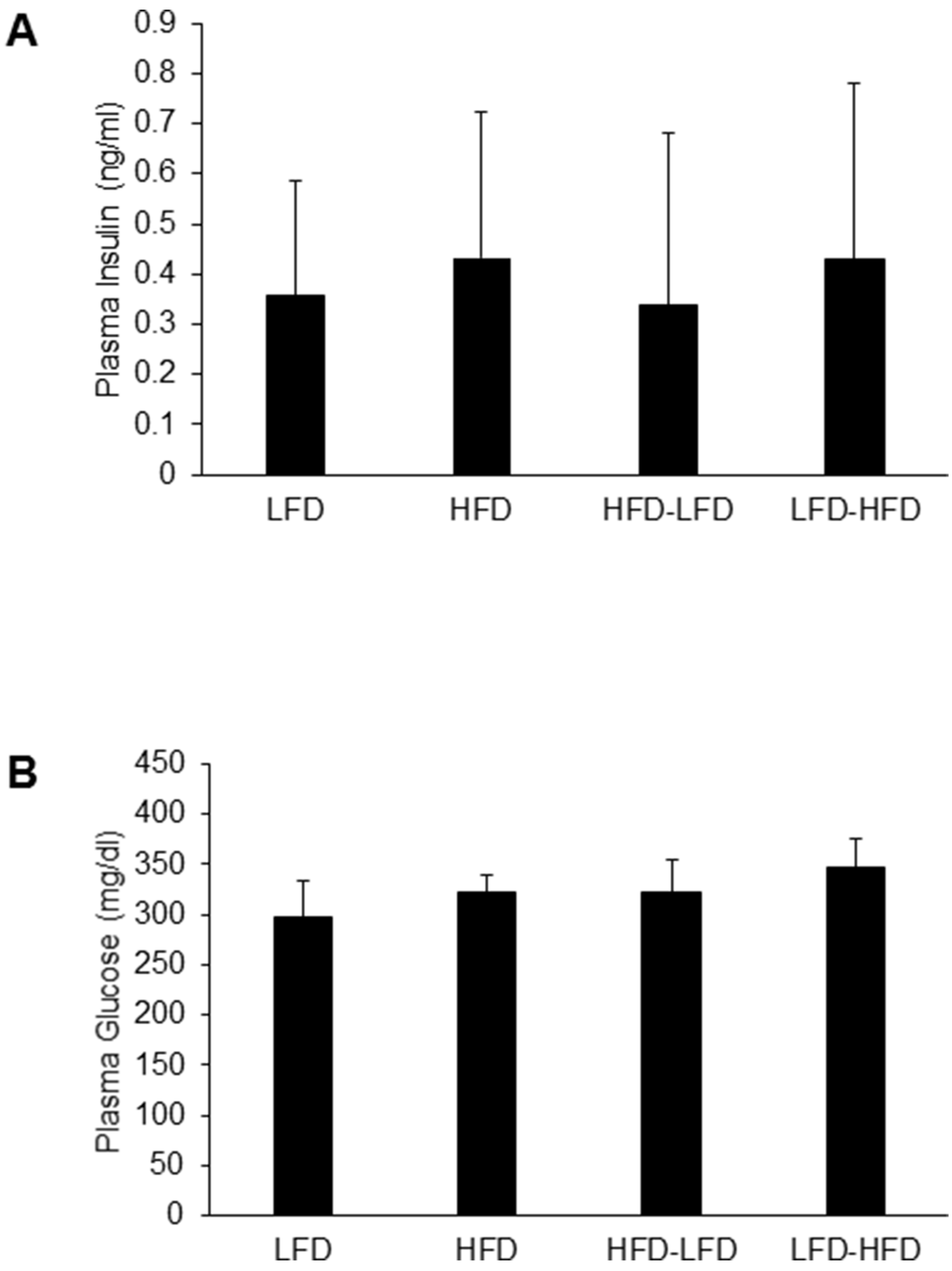

Supplementary Figure S2: Effects of dietary regimens on plasma insulin and glucose levels in 19-week old mice. No significant differences were observed among the dietary regimens. LFD (n=7); HFD (n=8); HFD-LFD (n=7); LHF-HFD (n=8).

Supplementary Table S1: Proportion of ER+/PR+ tumors by diet treatment and histopathology

Diet	Tumor Type	ER+PR+/Total
LFD	Epithelial	3/12
	Spindle Cell	0/5
HFD	Epithelial	8/26
	Spindle Cell	0/8
LFD-HFD	Epithelial	5/19
	Spindle Cell	0/12
HFD-LFD	Epithelial	5/21
	Spindle Cell	0/8

Supplementary Table S2: ANOVA of the effects measured versus diet, early and late onset epithelial tumors plus spindle cell tumors, and the interaction of diet with onset and/or spindle cell properties

Effect	Tumor Types	Diet	Onset/Spindle	Interaction
PCNA	Epi + Spindle	0.00	0.86	0.17
	Epi	0.01	0.89	0.01
TUNEL	Epi + Spindle	0.03	0.39	0.23
	Epi	0.15	0.89	0.09
CD31	Epi + Spindle	0.00	0.03	0.89
	Epi	0.02	0.42	0.72
F4/80	Epi + Spindle	0.03	0.35	0.66
	Epi	0.01	0.48	0.09
F4/80+Arg1	Epi + Spindle	0.00	0.20	0.83
	Epi	0.00	0.50	0.3

ANOVA was performed on data derived from epithelial and spindle cell tumors (Epi + Spindle) or epithelial tumors alone (Epi).

Supplementary Table S3: Gene ontology analysis comparing early tumors to late tumors

See Supplementary File 1

Supplementary Table S4: Mammary gland transplantation and occurrence of mammary tumors

	LFD	HFD	LFD-HFD	HFD-LFD
**Mice**	33	31	31	31
**Successful Transplants**	37	46	39	42
**Unsuccessful Transplants**	26	14	20	16
**Dead**	3	2	3	4
**Mammary Tumors**	17	30	27	26
**Early Tumors**	6	13	14	16
**Late Tumors**	11	17	13	12

For each diet group, the number of mice, successful mammary gland transplants, unsuccessful mammary gland transplants, glands unevaluated because of death, mammary tumors, early tumors (<52 weeks), and late tumors (≥52 weeks) are enumerated.

Supplementary Table S5: Diet composition

**Ingredients (g/100 g)**		**Low Fat Diet**	**High Fat Diet**
Fat	Corn Oil	2.369	16.1498
	Lard	1.8957	31.6537
Carbohydrate	Corn Starch	54.407	8.888
	Maltodextrin	11.848	16.1498
Protein	Casein	18.987	25.8397
	L-cystine	0.2843	0.3876
Fiber	Cellulose	4.7393	6.4599
Vitamins	Vitamin Mix V10001	0.9479	1.2919
	Choline Bitartrate	0.1896	0.2584
Minerals	Mineral Mix S10026	0.9479	0.1286
	DiCalcium Phosphate	1.2322	1.6795
	Calcium Carbonate	0.5213	0.7106
	Potassium Citrate, 1 H20	1.5639	2.1318
**Energy**			
kcal density/g		3.8	5.2
% kcal	Fat	10	60
	Carbohydrate	70	20
	Protein	20	20

Supplementary Table S6: BALB/c *Trp53-/-* tumors used for microarray analysis

Diet	Tumor#	Latency (weeks)
**Epithelial tumors**		
LFD-Early	31R	36
	22R	38
	17R	41
	11R	47
LFD-Late	13L	53
	19R	54
	27L4A	56
	11L	58
HFD-Early	42L4B	39
	60L	41
	65R	41
	40R4A	44
HFD-Late	57R	52
	66L	55
	56R	55
	62L	59
LFD-HFD-Early	80L	35
	92R	36
	78R	42
	82L	42
LFD-HFD-Late	71L	52
	101R	57
	101L4A	57
	90R	60
HFD-LFD-Early	117L	35
	115R	37
	117R	40
	107L	43
HFD-LFD-Late	108R	56
	109L	57
	110R	60
	122R	60
**Spindle Cell Tumors**		
LFD	33R	52
	18L	52
	32L	55
HFD	63L	36
	54R	52
	56LA	58
LFD-HFD	84R	39
	86R	46
	76R	60
HFD-LFD	128L	35
	129L	41
	118RA	52
