# Supplementary material for: Pubertal and adult windows of susceptibility to a high animal fat diet in Trp53-null mammary tumorigenesis
Source: Oncotarget. 2016 Nov 4;7(50):83409–23. doi: 10.18632/oncotarget.13112 (PMC5347778; doi:10.18632/oncotarget.13112)
Supplement: Supplementary file 2 [file oncotarget-07-83409-s002.doc]

**Table S3. Gene ontology analysis comparing early tumors to late tumors.**

**Down-Regulated Molecular Functions**

| **Categories** | **Function Annotation** | **B-H Adjusted p-Value** | **Molecules** | **No. of Molecules** |
| --- | --- | --- | --- | --- |
| Antimicrobial Response, Inflammatory Response | antiviral response | 1.58E-03 | IFIT1B,IFIT2,IFITM3,IL12B | 4 |
| Cellular Movement, Hematological System Development and Function, Immune Cell Trafficking | cell movement of myeloid cells | 4.68E-02 | CCL25,IL12B,IL2RB,Irgm1 | 4 |
| Cellular Movement, Hematological System Development and Function, Immune Cell Trafficking, Inflammatory Response | cell movement of macrophages | 4.15E-02 | CCL25,IL12B,Irgm1 | 3 |
| Endocrine System Disorders, Gastrointestinal Disease, Immunological Disease, Metabolic Disease | insulin-dependent diabetes mellitus | 2.89E-05 | GBP6,IFIT1B,IFIT2,IL12B,IL2RB,Irgm1,RNASE6,Trim30a/Trim30d | 8 |
| Inflammatory Response | inflammation of intestine | 4.68E-02 | CCL25,IL12B,PIP | 3 |
| Lymphoid Tissue Structure and Development, Organ Morphology, Tissue Morphology | abnormal morphology of lymph node | 3.32E-02 | CCL25,IL2RB,PIP | 3 |

**Up-Regulated Molecular Functions**

| Categories | Function Annotations | B-H Adjusted p-Value | Molecules | No. of Molecules |
| --- | --- | --- | --- | --- |
| Antimicrobial Response, Inflammatory Response | antimicrobial response | 4.06E-03 | Defa3 (includes others),DHX58,IRF7,Mx1/Mx2,RSAD2,SLAMF8,STAT1 | 7 |
|  | antiviral response | 5.59E-03 | DHX58,IRF7,Mx1/Mx2,RSAD2,STAT1 | 5 |
| Cell Death and Survival | killing of cells | 2.13E-02 | ADAR,CCR5,ITGAE,STAT1,TNFSF10 | 5 |
| Cell Signaling | replication of viral replicon | 4.06E-03 | ADAR,Mx1/Mx2,RSAD2 | 3 |
| Cellular Development, Skeletal and Muscular System Development and Function, Tissue Development | differentiation of myoblasts | 3.19E-02 | CXCL10,Cxcl9,Prl2c2 (includes others),STAT1 | 4 |
| Cellular Function and Maintenance, Hematological System Development and Function | function of T lymphocytes | 4.51E-02 | CCR5,CXCL10,HSH2D,IRF7,NLRC5,TNFSF10,Trim30a/Trim30d | 7 |
|  | function of lymphocytes | 4.52E-02 | CCR5,CXCL10,HSH2D,IRF7,NLRC5,STAT1,TNFSF10,Trim30a/Trim30d | 8 |
| Endocrine System Disorders, Gastrointestinal Disease, Immunological Disease, Metabolic Disease | insulin-dependent diabetes mellitus | 8.51E-14 | CCR5,CXCL10,Cxcl9,GBP6,Gbp8,Gm5431,HERC6,HPSE,IFI16,IFI44,Ifi47,Iigp1,IRF7,ITGAE,PARP14,STAT1,STAT2,Tgtp1/Tgtp2,TNFSF10,TNFSF8,Trim30a/Trim30d | 21 |
| Endocrine System Disorders, Gastrointestinal Disease, Metabolic Disease | diabetes mellitus | 9.18E-13 | CCR5,CXCL10,Cxcl9,GBP6,Gbp8,Gm5431,HERC6,HPSE,IFI16,IFI44,Ifi47,Iigp1,IRF7,ITGAE,LGALS9B,PARP14,STAT1,STAT2,Tgtp1/Tgtp2,TNFSF10,TNFSF8,Trim30a/Trim30d | 22 |
| Hematological Disease | toxemia | 1.94E-02 | CCR5,CXCL10,Cxcl9,STAT1,Trim30a/Trim30d | 5 |
| Immunological Disease | systemic autoimmune syndrome | 9.18E-13 | CCR5,CXCL10,Cxcl9,GBP6,Gbp8,Gm5431,HERC6,HPSE,IFI16,IFI44,Ifi47,IGFBP4,Iigp1,IRF7,ITGAE,PARP14,STAT1,STAT2,Tgtp1/Tgtp2,TNFSF10,TNFSF8,Trim30a/Trim30d | 22 |
| Infectious Disease | Viral Infection | 5.46E-05 | ADAR,CCR5,CXCL10,DHX58,IRF7,LGALS9B,Mx1/Mx2,RSAD2,STAT1,STAT2,TNFSF10 | 11 |
|  | replication of virus | 2.45E-03 | CCR5,CXCL10,Mx1/Mx2,RSAD2,STAT1,TNFSF10 | 6 |
|  | infection of mammalia | 2.95E-03 | BAHD1,CCR5,CXCL10,DHX58,Ifi47,Iigp1,IRF7,ITGAE,STAT1,STAT2,TNFSF10 | 11 |
|  | anthrax | 4.06E-03 | CXCL10,Cxcl9,STAT1 | 3 |
|  | Bacterial Infection | 3.19E-02 | BAHD1,CCR5,CXCL10,Cxcl9,IRF7,STAT1,TNFSF10,Trim30a/Trim30d | 8 |
|  | replication of RNA virus | 3.48E-02 | CCR5,CXCL10,Mx1/Mx2,RSAD2 | 4 |
| Infectious Disease, Respiratory Disease | infection of lung | 3.19E-02 | CXCL10,Cxcl9,STAT1 | 3 |
| Metabolic Disease | glucose metabolism disorder | 9.85E-09 | BGLAP,CCR5,CXCL10,Cxcl9,GBP6,Gbp8,Gm5431,HERC6,HPSE,IFI16,IFI44,Ifi47,Iigp1,IRF7,ITGAE,LGALS9B,PARP14,STAT1,STAT2,Tgtp1/Tgtp2,TNFSF10,TNFSF8,Trim30a/Trim30d | 23 |
| Organismal Survival | survival of organism | 4.52E-02 | CCR5,CXCL10,Cxcl9,DHX58,IFI16,IRF7,Mx1/Mx2,RSAD2,SMN1/SMN2,STAT1,STAT2,TNFSF10 | 12 |
| Protein Synthesis | quantity of interferon | 1.94E-02 | ADAR,DHX58,RSAD2,STAT1 | 4 |
